# Improving Health and Well-Being of People With Post–COVID-19 Consequences in South Africa: Situation Analysis and Pilot Intervention Design

**DOI:** 10.2196/58436

**Published:** 2025-04-10

**Authors:** Nicole Audrey Glover, Farzana Sathar, Pride Mokome, Nkululeko Mathabela, Sipokazi Taleni, Sarah Alexandra van Blydenstein, Anna-Maria Mekota, Salome Charalambous, Andrea Rachow, Olena Ivanova

**Affiliations:** 1 Implementation Research Division, The Aurum Institute, 29 Queens Road, Parktown, Johannesburg, 2193, South Africa, 27 72 277 2261; 2 Department of Internal Medicine, Chris Hani Baragwanath Academic Hospital, University of the Witwatersrand, Johannesburg, South Africa; 3 Institute of Infectious Diseases and Tropical Medicine, Ludwig-Maximilians-Universität University Hospital, Munich, Germany; 4 School of Public Health, University of the Witwatersrand, Johannesburg, South Africa; 5 German Research Centre for Environmental Health, Helmholtz Zentrum München, Unit Global Health, Neuherberg, Germany; 6 German Center for Infection Research, Partner Site Munich, Munich, Germany

**Keywords:** post–COVID-19, rehabilitation, support, quality of life, group care, well-being, South Africa, COVID-19, situation analysis, pilot, intervention, context-adapted, physical health, mental health, cross-sectional, mixed method, questionnaire, in-depth, interviews, survey, focus group, quantitative, qualitative, support group, hospital, patients, health care workers, health worker

## Abstract

**Background:**

Multisystemic complications post–COVID-19 infection are increasingly described in the literature, yet guidance on the management remains limited.

**Objectives:**

This study aimed to assess the needs, preferences, challenges, and existing interventions for individuals with post–COVID-19 symptoms. Based on this, we aimed to develop a context-adapted intervention to improve the overall health and well-being of individuals with post–COVID-19 complications.

**Methods:**

We conducted a cross-sectional mixed-methods situation analysis assessing the needs, preferences, challenges, and existing interventions for patients with post–COVID-19 symptoms. We collected data through questionnaires, semistructured in-depth interviews, and focus group discussions (FGDs) from individuals diagnosed with COVID-19 within the previous 18-month period and health care providers who managed patients with COVID-19 in both inpatient and outpatient settings. Quantitative data were summarized using descriptive statistics, qualitative data were transcribed, and deductive analysis focused on suggestions for future interventions. Findings guided the development of a group intervention.

**Results:**

We conducted 60 questionnaires, 13 interviews, and 3 FGDs. Questionnaires showed limited knowledge of post–COVID-19 complications at 26.7% (16/60). Of those who received any rehabilitation for COVID-19 (19/60, 31.7%), 94.7% (18/19) found it helpful for their recovery. Just over half (23/41, 56%) of those who did not receive rehabilitation reported that they would have liked to. The majority viewed rehabilitation as an important adjunct to post–COVID-19 care (56/60, 93.3%) and that support groups would be helpful (53/60, 88.3%). Qualitative results highlighted the need for mental health support, structured post–COVID-19 follow-up, and financial aid in post–COVID-19 care. Based on the insights from the situation analysis, the theory of change framework, and existing post–COVID-19 evidence, we designed and conducted a pilot support group and rehabilitation intervention for individuals with post–COVID-19 complications. Our main objective was to assess the change in physical and psychological well-being pre- and postintervention. The intervention included 8 weekly themed group sessions supplemented by home tasks. Effectiveness of the intervention was evaluated by questionnaires pre- and postintervention on post–COVID-19 symptoms, quality of life with the EuroQoL 5-Dimension 5-Level, short Warwick-Edinburgh Mental Wellbeing Scale, and physical function by spirometry and 1-minute sit-to-stand test. We also assessed the feasibility and acceptability of the intervention by questionnaires and semistructured in-depth interviews. The intervention outcome analysis is yet to be conducted.

**Conclusions:**

Insights from patients and health care providers on the characteristics of post–COVID-19 complications helped guide the development of a context-adapted intervention program with potential to improve health and well-being post–COVID-19.

## Introduction

Most people who have had SARS-CoV-2 infection, causing COVID-19 disease, recover fully; however, a proportion experience longer-term complications resulting in persistent symptoms and functional impairment [1,2]. Terms used to describe this syndrome include long COVID-19, post-acute COVID-19 syndrome, or post–COVID-19 condition; however, the definitions remain heterogeneous [1-3]. Post–COVID-19 complications can affect multiple body systems including pulmonary, cardiovascular, neurological, and musculoskeletal systems, where over 50 long-term symptoms have been identified [1,4]. The prevalence of post–COVID-19 complications varies substantially, from 6% up to 80%, depending on definitions used, hospitalization status, and duration of follow-up [2,4,5]. Common symptoms include fatigue, headache, arthralgia and myalgia, dyspnea and notably neurocognitive, mental health, and sleep disorders [2,6,7]. In addition to the symptoms, objective assessments of lung, cardiac, and neurological function demonstrate long-term pathology [1,2,8]. These complications are reported to impact activities of daily living (ADL), quality of life, and ability to work [9,10].

Limited data exists in African countries on post-COVID-19 morbidity and its characterization [2,4,6]. A study conducted in South Africa demonstrated COVID-19-related symptoms in 66.7% of people 3 months posthospital discharge [11], which was supported in another study by Jassat et al [12]. Further descriptions of post–COVID-19 complications in the South African context have come from our previous study, demonstrating pathology in hospitalized post–COVID-19 individuals, where up to 1-month posthospital discharge 85.2% had abnormal chest–computed tomography, 49.5% had abnormal spirometry, 79% had abnormal diffusing capacity of the lung, and functional limitation was evidenced by reduced median 6-minute walking test (300, IQR 210-400 m) and elevated St George’s Respiratory Questionnaire scores (median 21.6, IQR 9.2-48.3) [13]. Despite these findings, sample sizes are small and the breadth of data and characterization of post–COVID sequelae in these cohorts is limited.

In addition to the multisystem sequelae recognized post–COVID-19, recovery can be complicated by preexisting or newly developed chronic diseases such as cardiovascular disease, diabetes, HIV, and tuberculosis (TB) [14]. Understanding these multisystem complications is integral to directing health services effectively [9]. Due to the complexity of recovery post–COVID-19, a holistic approach should be taken for management. Furthermore, the burden of disease and local health resources differ across regions [2,6], demanding the evaluation of context-specific assessment tools and interventions, to improve long-term recovery post–COVID-19 [15-17].

The World Health Organization (WHO) and National Institute for Health and Care Excellence (NICE) guidelines recommend multidisciplinary and patient-centered care for post–COVID-19 complications [3,18]. In a randomized control trial conducted by Liu et al [19], a respiratory rehabilitation program was shown to improve respiratory function, quality of life, and anxiety. These findings are reflected in another study where post–COVID-19 rehabilitation showed improvement in lung function and a reduction in anxiety levels [20].

Considering this multidisciplinary approach in resource-constrained settings, such as low- and middle-income countries (LMICs), assisted self-management programs and peer support groups become pertinent. This was evidenced by Wright et al [21], where a self-management program guided by weekly educational support sessions demonstrated improvement in patient self-efficacy and mental well-being post–COVID-19 [21,22]. Self-management techniques and patient education with other chronic lung diseases, including post-TB lung disease and bronchiectasis, have been noted to empower patients, improve patient confidence, competence, and overall quality of life [23,24]. However, few studies are available on context-adapted and culturally appropriate tools and interventions for the management of post–COVID-19 in LMICs, the importance of which was highlighted by Jassat et al [25].

This paper aims to describe the findings from a situation analysis on post–COVID-19 rehabilitation services and the development of a context-adapted pilot intervention aimed to improve overall physical and mental health post–COVID-19 conducted in Johannesburg, South Africa.

## Methods

Both the situation analysis and intervention have been conducted. Below we outline the methods used for the situation analysis and framework for intervention development.

### Situation Analysis

We conducted a mixed-method situation analysis study with our main aim to explore the post–COVID-19 management landscape by assessing the needs, preferences, challenges, and existing interventions and strategies for patients with post–COVID-19 complications in Johannesburg, South Africa.

We conducted this study at the Chris Hani Baragwanath Academic Hospital (CHBAH) and Tembisa Provincial Tertiary Hospital (Tembisa), Johannesburg, South Africa. These health care facilities are located in townships in the City of Johannesburg and Ekurhuleni districts and cater to the general population. They are public health care facilities funded by the Gauteng Department of Health. Participants were recruited from outpatient medical and rehabilitation departments at the respective facilities, where patients who had COVID-19 disease were invited to participate, and health care workers who had managed patients with COVID-19 were invited to participate. Potential participants were approached in the waiting areas of clinics, where prescreening procedures assessing COVID-19 history were done followed by full informed consent if potential participants were willing. The study was conducted between December 2022 and February 2023, which was 11-months after the last clear reported COVID-19 wave in South Africa [26].

We collected quantitative data through a cross-sectional standardized questionnaire-based interview (see Multimedia Appendix 1) administered by the research staff with individuals who were previously diagnosed with COVID-19. The questionnaire covered topics on post–COVID-19 symptoms, experience with rehabilitation programs, challenges, preferences, and suggestions for potential interventions. A total of 2 research assistants (male and female) and a research nurse (female) were trained and collected the survey data. We used descriptive statistics to summarize the data in frequencies, mean and median, using Microsoft Excel.

The qualitative study included semistructured in-depth interviews with health care workers who manage COVID-19 patients and focus group discussions (FGDs) with individuals who had post–COVID-19 symptoms. Separate interviews were used with health care workers to allow in-depth conversation on each discipline, and FGDs were used with patients to allow a broad conversation flow of COVID-19 reflection and to encourage group sharing (see MultimediaAppendices 2  3). Interviews and FGDs were facilitated by the research team (3 females and 1 male), in English, except 2 FGD’s conducted in a combination of English, isiZulu, and Sepedi. The duration of the interviews and FGDs ranged from 10-minutes to 90-minutes depending on participant availability and willingness to engage in the content of the questions. A guide was used for the interviews focusing on health care worker reflection on managing COVID-19 patients, challenges and perceived need for rehabilitation; and a guide was used in the FGDs to discuss patient post–COVID-19 experiences and rehabilitation needs and challenges. Both guides allowed for flexibility of the discussion. Interviews and FGDs were recorded, transcribed, coded, and deductive thematic analysis was carried out focusing on main concepts for the intervention development.

Quantitative and qualitative data were triangulated through an iterative process to ensure the coherence of findings and obtain more insights on the desirable content and format of the intervention.

### Intervention Development

The creation of the intervention followed the steps described in the Group Intervention framework [27]. We ensured a suitable context and setting for the intervention. Following this, we designed the group intervention addressing outcomes and measurements, sampling into groups, quantity of groups, and structure of the intervention. Finally, during the delivery of the intervention, we introduced team briefings and group notes to describe and reflect on the dynamics within each group and session, position of group participants and leaders, and communication.

The data collected in the situation analysis and available evidence in post–COVID-19 rehabilitation fed into the content and delivery format of the intervention. The design process, implementation, and evaluation plan for the intervention are described in more detail in the Results section. The outcomes of the intervention are being analyzed and will be presented in a separate paper.

### Ethical Considerations

We obtained ethical approval for the situation analysis from Wits Human Research Ethics Committee (230405), and the 2 facilities, CHBAH and Tembisa Hospital. All participants gave written informed consent before participation. The study data are deidentified to protect participant confidentiality. Participants received R 300 (US $16.85) cash reimbursement for each study visit covering costs for travel and time.

## Results

### Situation Analysis and Needs Assessment

#### Quantitative Results

We conducted 60 questionnaires with individuals who had COVID-19; Table 1 shows the findings. We had 21.7% (13/60) male participants, with a median age of 49.5 (IQR 39.5-60) years. Knowledge of post–COVID-19 sequelae was limited, with only 26.7% (16/60) having heard of these complications. From the 60 participants, 31.7% (19/60) received any rehabilitation for COVID-19 in either inpatient or outpatient setting, where physiotherapy was the most common (17/19, 89.5%), followed by dietician (4/19, 21.1%), speech therapy and audiology (3/19, 15.8%), psychology (2/19, 10.5%), and occupational therapy (1/19, 5.3%). Of those who received rehabilitation, all received rehabilitation within 6 weeks of COVID-19 infection and 94.7% (18/19) found it helpful for recovery. Of those who did not receive any rehabilitation, 56.1% (23/41) reported that they would have liked to receive rehabilitation, notably psychological support and counseling (14/23, 60.9%) and physiotherapy (8/23, 34.8%). Majority of the participants felt that rehabilitation was an important adjunct to COVID-19 recovery (56/60, 93.3%) and said that they would find a support group helpful for their recovery post–COVID-19 (53/60, 88.3%).

**Table 1.** Characteristics and questionnaire responses from participants (N=60). Quantitative data collected in situation analysis at Chris Hani Baragwanath Academic Hospital and Tembisa Provincial Tertiary Hospital from December 2022 to February 2023.

Characteristic	Participants (N=60)
Sex (male), n (%)	13 (21.7)
Age (years), median (IQR)	49.5 (39.5-60)
Knowledge of post–COVID-19 complications^a^, n (%)	16 (26.7)
Received rehabilitation for acute COVID-19 or post–COVID-19 complications, n (%)	19 (31.7)
Inpatient rehabilitation	14 (73.7)
Outpatient rehabilitation	5 (26.3)
Physiotherapy	17 (89.5)
Dietician	4 (21.1)
Speech therapy and audiology	3 (15.8)
Psychology	2 (10.5)
Occupational therapy	1 (5.3)
Received rehabilitation for acute COVID-19 or post–COVID-19 complications and found it helpful for recovery, n (%)	18 (30)
Did not receive rehabilitation and would have liked to, n (%)	23 (38.3)
Psychology or counseling requested	14 (23.3)
Physiotherapy requested	8 (13.3)
Main goals for recovery post–COVID-19, n (%)	
To carry out activities of daily living better	34 (56.7)
To be able to exercise or have better physical capacity	32 (53.3)
Improved mental health or coping strategies	25 (41.7)
Return to work	21 (35)
Connection with people and to be able to care for family	20 (33.3)
Feel rehabilitation is an important adjunct to care post–COVID-19, n (%)	56 (93.3)
Would find a support group helpful for recovery post–COVID-19, n (%)	53 (88.3)

a Knowledge of post–COVID-19 complications was assessed by asking “Do you know what long-COVID or post-COVID-19 condition is?” and allowing participants to answer yes or no and elaborate on their answer.

#### Qualitative Results

We conducted 13 interviews with health care providers and 3 FGDs with a total of 20 post–COVID-19 patients. The 3 main themes that emerged from the thematic analysis for potential interventions post–COVID-19 included the need for mental health support, structured post–COVID-19 follow-up, and the need for financial aid.

Need for mental health support was further explained by the request to focus on psychological well-being and support groups. Both health care providers and patients reported that psychological well-being was neglected during the pandemic and should be a focus in post–COVID-19 care. One participant reported:

*I think we focus more on the physical healing than the psychological healing. So, most people who had Covid, they are not okay. They had post-Covid depression or post-Covid anxiety. So, I think that there is a lot of mental health [care] that needs to be done post-Covid.*

[Female, health care provider]

Health care providers and patients who had opportunities to have debriefing sessions and support groups, further expanded on the importance of incorporating support groups into the mental health support post–COVID-19:

*They really must get help. From our side… sitting together talking, [about our experience], it’s a good thing. It’s a helping thing, it’s a very, very good thing. Thank you.*

[Male, patient in FGD]

This importance of sharing experiences and leaning on additional support from others in the group context during post–COVID-19 care was further supported by a health care provider, saying:

*… looking at a rehab unit where… people feel comfortable with coming in… sharing their stories to each other, and their success stories, because that’s how you then motivate each other. It’s not coming from someone that may not understand it, you’re hearing it from patient to patient. And that might be nice.*

[Female, health care provider]

In addition to the mental health support, structured medical post–COVID-19 follow-up care was also reported as an important part of post–COVID-19 care, where both patients and health care providers should have a structured follow-up program, rehabilitation, and referral to specialists depending on their complications. The importance of recognizing each individual and the treatment that they require was emphasized:

*We are not statistics, we are human beings, we are sick, and we are affected by COVID, and I think there must be a way to create a rehab for us [to have] proper check-up for us, because she is telling [us] about her lungs and I am telling you about my eyes and what are they doing? So, in six months or one year you must take this person to a specialist for check-ups.*

[Female, health care provider]

Finally, financial aid was recognized as an important area for support post–COVID-19, largely expressed by patients and recognized by health care workers. Patients reported that their daily functioning is worse compared with their functioning before COVID-19. Financial assistance was requested for those who can no longer work due to their complications post–COVID-19:

*… he said he is no longer going to work. His salary is no longer the same and also our sister here, she her health is no longer the same. I think they are supposed to approve her for grant.*

[Female, patient in FGD]

Taking into account our findings from both the quantitative and qualitative data, there was a strong emphasis on mental health support, support groups, and structured medical and rehabilitation follow-up post–COVID-19.

### Design and Implementation of the Intervention

**Figure 1.**
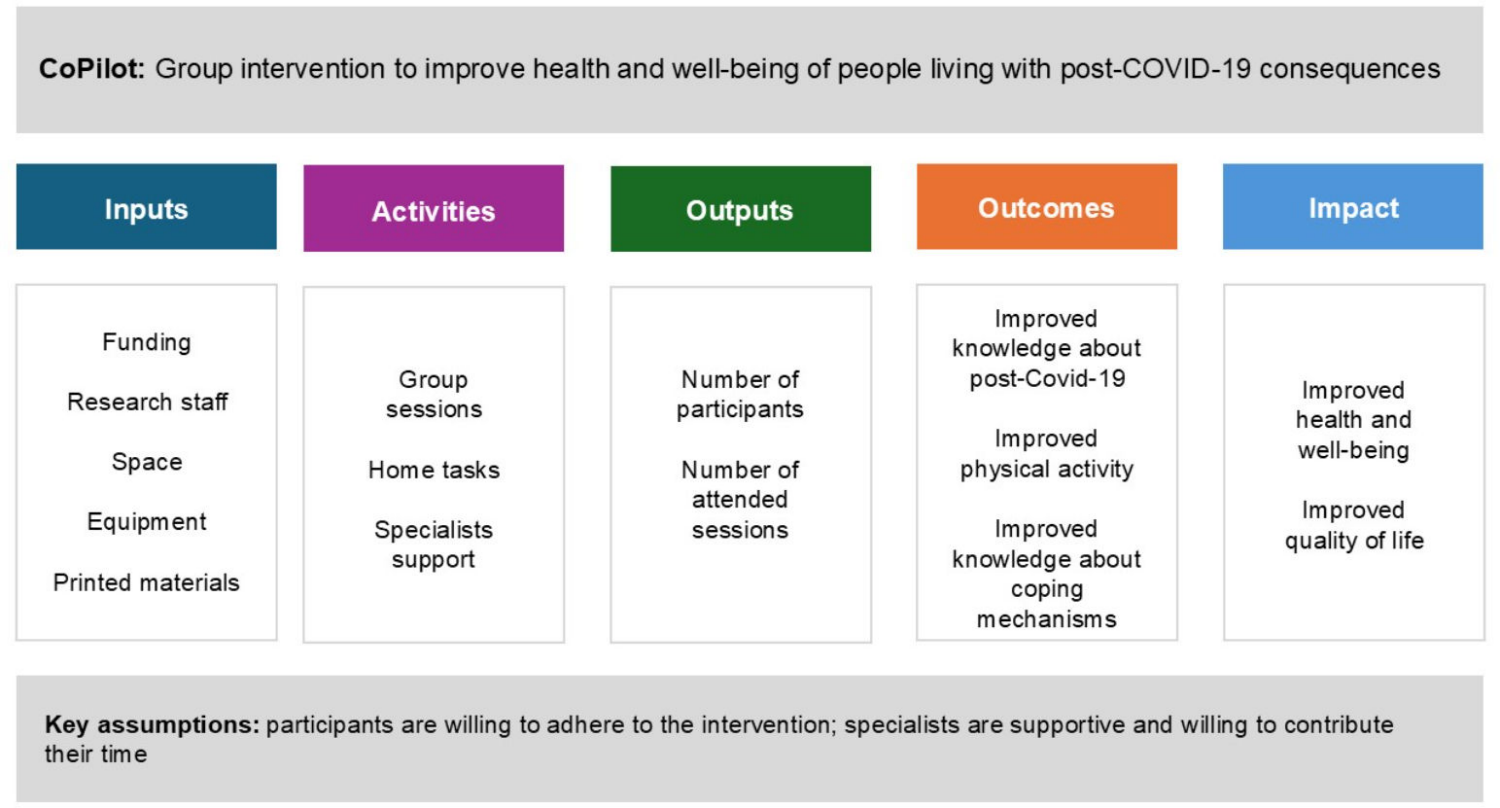
Theory of change for CoPilot, demonstrating the post–COVID-19 intervention development plan adapted to the theory of change framework for individuals with post–COVID-19 complications.

Using the information obtained from the situation analysis and existing evidence on post–COVID-19 rehabilitation, we developed an in-person support group program for people with post–COVID-19 complications titled CoPilot. The main study objective was to assess the change in physical and psychological well-being in patients with post–COVID-19 sequelae before and after the pilot intervention. To guide the program and evaluation we present a theory of change (Figure 1). The main intended impact of the intervention is to improve the overall health and well-being of COVID-19 survivors.

The target population for the intervention was individuals who had been diagnosed with COVID-19 and were experiencing ongoing post–COVID-19 complications according to the WHO post–COVID-19 condition definition [3]. The participants were recruited from out-patient medical and rehabilitation departments at the health care facility, including both patients and health care providers, and further participants were recruited by snowball sampling. Eligibility criteria included: self-reported or diagnosed previous COVID-19; adults aged 18-years and older; reporting at least one symptom lasting for more than 2 months, at least 3 months since COVID-19, which could not otherwise be explained by another condition; and willingness to provide written informed consent and to follow study procedures. Reported symptoms could include, but were not limited to, fatigue, breathlessness, pain or discomfort, impaired sleep, or impaired usual activity [2]. Participants with known severe medical or psychiatric conditions which affected ability to consent or participate, or required a higher level of care were excluded.

Group formats in rehabilitation have been shown to strengthen curative factors like universality and altruism, to provide peer and social support, to share coping strategies, and to practice gratitude [28,29]. Taking this into account, with the strong findings for group support in post–COVID-19 rehabilitation from our situation analysis, we designed and conducted a group-based intervention. The program consisted of weekly in-person group sessions over eight-weeks complimented by home-tasks and self-management exercises, with the option to adapt tasks to each person’s abilities. We had 7 groups where each group consisted of 8 to 10 participants. Our target sample size was 60, which was informed by evidence that sample sizes of 24‐50 are sufficient to estimate the key parameters of an efficacy and feasibility trial [30]. Sessions were guided by the predesigned schedule and a duration of one-hour, but with flexibility for participant experiences and needs. The core of the sessions involved research staff facilitating discussion around participants’ post–COVID-19 experiences, challenges and self-management strategies whilst encouraging group support and education on post–COVID-19 complications. Select sessions included invited rehabilitation specialists (eg, psychologist, physiotherapist, and dietician) for additional expertise. If any participant missed a session, they were contacted telephonically to explain the weekly tasks. If participants missed a session and were not contactable, they were regarded as lost-to-follow-up.

The content of the intervention was guided by the findings from our situation analysis, the WHO Brochure “Support for rehabilitation: self-management after COVID-19-related illness [31] and from the HOPE intervention for people with post–Covid-19 conducted by Wright et al [21]. The detailed description of the session’s content, weekly themes, evidence that informed the themes are shown in Table 2 along with the planned duration of the session and home-tasks.

**Table 2.** Intervention modules and content.

Module	Content	Evidence-informing theme	Format: 1 hour	Home-task
Week 1: post–COVID-19 complications introduction	Welcome and introductions. Overview of the program. Pacing and goal setting. Breathing exercise introduction.	Quantitative findings on lack of post–COVID-19 knowledge. HOPE intervention^a^. Supported by WHO^b^ brochure.	Group session facilitated by research staff – discussions, home tasks.	Self-test: how are you feeling? Goal setting: SMARTER tool (general)^c^. Long–COVID-19 education. Breathing exercise.
Week 2: symptoms and physical activity	Main symptom complaints. Exercise routine and demonstration. Setting physical goals. Share experience and symptom management. Fatigue management.	Quantitative findings on lack of post-COVID-19 knowledge, and goals for post-COVID-19 recovery to be able to carry out activities of daily living better and have better physical capacity. Qualitative finding on providing post-COVID-19 symptom follow-up. Supported by HOPE intervention and WHO brochure.	Group session with physiotherapist – discussion, exercise demonstration, home tasks.	Exercises self-moderated. SMARTER goal setting (physical ability focus).
Week 3: coping strategies	Prioritizing and taking breaks. Relaxation. Mindfulness practices to manage stress.	Quantitative findings on request for psychological support, goal for post–COVID-19 recovery of better coping strategies. Qualitative findings on need for mental health support.	Group session facilitated by research staff – discussions, guided meditation.	SMARTER goal setting (relaxation focus). Relaxation and mindfulness tasks. Gratitude diary.
Week 4: support and grief	Debriefing. Grief process and tracker. Finding support. Self-compassion and prioritization. Asking for and accepting help.	Quantitative findings on request for psychological support, goal for post–COVID-19 recovery of better coping strategies, and better connection with people and family. Qualitative findings on need for mental health support.	Group session with psychologist – discussion and home tasks.	Grief tracker. Speaking to family or a friend. Self-compassion task. Self-test: how are you feeling?
Week 5: sleep and mindfulness	Sleep disorder information. Tips for better sleep. Mundane task focusing.	Quantitative finding of goal for post–COVID-19 recovery for better coping strategies. Supported by HOPE intervention.	Group session facilitated by research staff – discussions, home tasks.	Sleep diary. SMARTER goal setting (sleep). Mundane task focusing.
Week 6: move and eat better	Importance of keeping active. Eating well for physical and mental health. Continuing exercises.	Quantitative findings on goals for post–COVID-19 recovery to be able to carry out activities of daily living better and have better physical capacity. Supported by HOPE intervention and WHO brochure.	Group session with dietician – discussion and home tasks.	SMARTER goal setting (nutrition). Home exercises and stretching. What makes you happy?
Week 7: happiness and strengths	Long COVID-19 and mental health education. Identifying strengths and how to use them. Managing setbacks. Reviewing, what makes you happy?	Quantitative findings on request for psychological support, goal for post–COVID-19 recovery of better coping strategies. Qualitative findings on need for mental health support.	Group session facilitated by research staff – discussions and home tasks.	Gratitude diary. SMARTER goal setting (strengths and happiness). Long–COVID-19 and mental health education.
Week 8: looking forward	Hopes and aspirations. Keeping in touch with support. Review of the program. Planning pleasant activities.	Quantitative findings on request for psychological support, goal for post–COVID-19 recovery of better coping strategies, and better connection with family. Qualitative findings on need for mental health support. Supported by HOPE intervention.	Group session facilitated by research staff – discussions and home tasks.	Self-test: how are you feeling? SMARTER goal setting (hopes for the future). Moving forward with hope.

a HOPE intervention refers to Hope Programme for Long COVID-19 [21].

b WHO: World Health Organization.

c The SMARTER goal-setting tool is used to define goals following the criteria: specific, measurable, achievable, relevant, time-bound, exciting, and reviewable.

### Monitoring and Evaluation Plan

Monitoring of the intervention was done using a participant registry, discussions at each session to review understanding of the content and tasks, and completion of a questionnaire to assess adherence to tasks and subjective progress.

The evaluation of the intervention was performed by measuring effectiveness (pre-and postintervention assessment) and a process evaluation (including feasibility and acceptability) of the group intervention. The main effectiveness outcomes included: (1) lung function, (2) mental well-being, and (3) quality of life. Data collection included baseline information on demographics and knowledge of COVID-19 and sequelae. The pre-post-intervention assessments included questionnaires on COVID-19 symptoms, fatigue and sleepiness scales [21,32,33], quality of life using the EuroQoL 5-Dimension 5-Level (EQ-5D-5L) [34], short Warwick-Edinburgh Mental Wellbeing Scale to describe mental well-being [35], lung function testing by means of spirometry, and functional capacity by 1-minute sit-to-stand test [36]. The process evaluation was guided by the Medical Research Council framework to assess both the implementation process (how the project is delivered), what is delivered (fidelity, dose, adaptations, or reach) and contextual factors influencing the implementation and acceptability of the intervention [37]. We used mixed methods to assess feasibility and acceptability of the intervention through a questionnaire and semistructured interviews with participants to elaborate on the design of the intervention, the potential barriers, and facilitators. Data from the intervention will be analyzed using descriptive statistics producing frequencies, means, and proportions of the different variables of interest. Outcomes will be analyzed using paired sample *t* tests. Qualitative process evaluation data will be transcribed and analyzed by thematic analysis.

## Discussion

### Principal Findings

To the best of our knowledge, this is the first study to develop and implement a context-adapted group intervention for post–COVID-19 survivors in South Africa. It was aimed to address debilitating consequences of COVID-19 and provide people with the tools to manage their symptoms to improve every-day quality of life and potentially health outcomes including mental and lung health. This situation analysis and intervention development highlighted 2 main points: first, engaging with patients and health care workers on their perspectives on post–COVID-19 care is an integral component in developing management strategies for post–COVID-19 complications, and second, that mental health support should strongly be considered in rehabilitation guidelines post–COVID-19 while considering support groups as a component.

Engaging with health care providers and patients’ perspectives on post–COVID-19 complications and the management thereof is a crucial step in guideline development. The cornerstone to this is understanding the patient experience of post–COVID-19 complications and leveraging on existing and innovative strategies for care [21,25]. Fatigue is a common symptom reported post–COVID-19; however, it has been described by patients to have physical, mental, and emotional components [6,21], thereby illustrating that understanding the problem on a deeper level can help direct health services toward patient needs, as well as develop suitable assessment measures for patient-reported outcomes [9]. Understanding patient and health care provider perspectives and communication between them has been shown to be important in HIV care, specifically relating to social, mental health, and health care system challenges [38]. In addition, coworking and partnership with shared responsibility in care between health care provider and patient with a focus on self-management principles were recognized as key features in the management process of other chronic lung diseases [24]. Taking into account the health resource limitations in LMICs and the strain on insufficient rehabilitation services poses another challenge to the management of post–COVID-19 complications, emphasizing the need for context-adapted guidelines [39].

The importance of the need for mental health support was a major theme that came through during our situation analysis. Mental health symptoms have been reported in up to 21% of people post–COVID-19 and can include anxiety, depression, sleep disorders, and neurocognitive disorders [4,6]. In a study by Rofail et al [9], it was reported that despite no clear psychological diagnosis, most impacts on daily life were not attributed to any singular symptom, but rather to the holistic experience of COVID-19 infection, further supporting the need to support psychological processing in recovery. Findings from our situation analysis clearly showed the gaps in psychological support for patients and health care providers post–COVID-19 and that these services are sought after as an adjunct to post–COVID-19 care. More specifically, support groups were suggested as helpful platforms to engage in psychological support, facilitating connection and shared experiences. This focus in care has been shown to be valued and helpful in support of patients with other chronic lung conditions [24].

One limitation is that this was a narrow population, where participants were only recruited from 2 facilities; however, to overcome this we recruited from multiple departments and recruited both health care providers and patients in order to increase variation. Another limitation was the heterogeneity of the post–COVID-19 sequelae definition, where interpretation conducted by the study team was to meet the WHO post–COVID-19 condition definition. This could result in bias of either overestimating or underestimating post–COVID-19 consequences, depending on alternate definitions used in other contexts. Despite this, consistency was maintained throughout recruitment for the situation analysis and planned intervention, and symptoms were clearly documented.

The strengths of the study include the context and population of the study where post–COVID-19 rehabilitation has not been studied previously. Another strength includes the mixed-methods approach for both the situation analysis and intervention in order to obtain deeper insight into the rehabilitation post–COVID-19.

### Conclusion

Understanding the characteristics and burden of post–COVID-19 complications, particularly from the patient and health care provider perspectives, helps to direct post–COVID-19 management principles to ensure a more holistic recovery. As more people recover from COVID-19, it becomes crucial to maximize on the opportunity to better understand postviral complications and adapted health service allocation, particularly in LMICs. We believe that our group intervention holds a potential to improve health and well-being of people living with post–COVID-19 complications in Johannesburg, and its effectiveness and feasibility will be analyzed in the next steps.

## Supplementary material

10.2196/58436 Multimedia Appendix 1 Questionnaire guide.

10.2196/58436 Multimedia Appendix 2 Focus group discussion guide.

10.2196/58436 Multimedia Appendix 3 Clinical staff interview guide.

## Abbreviations

ADL: activities of daily living
CHBAH: Chris Hani Baragwanath Academic Hospital
EQ-5D-5L: EuroQoL 5-Dimension 5-Level
FGD: focus group discussion
LMIC: low- and middle-income country
NICE: National Institute for Health and Care Excellence
TB: tuberculosis
WHO: World Health Organization
