# Supplementary material for: Improving Health and Well-Being of People With Post–COVID-19 Consequences in South Africa: Situation Analysis and Pilot Intervention Design
Source: JMIR Form Res. 2025 Apr 10;9:e58436. doi: 10.2196/58436 (PMC12005461; doi:10.2196/58436)
Supplement: Multimedia Appendix 1 [file formative-v9-e58436-s001.docx]

| **No.** | **Question** | **Answer** |
| --- | --- | --- |
| 1.1 | Date of birth | \|__\|__\|/ \|__\|__\| /\|__\|__\|__\|__\|  if day or month are not known, write ***99.*** If year is not known, write ***9999*** |
| 1.2 | How old are you now? | \|__\|__\| years |
| 1.3 | Sex at birth |  Male   Female   Other |
| 1.4 | What is your highest level of education? |  Never attended school   Primary   High school   Matric   Tertiary   Other, specify ___________ |
| 1.5 | Occupation |  Full-time employee   Part-time employee   Self-employed   Pensioner   Unemployed   Informal work   Other, specify ____________ |
| 2.1 | When did you have Covid-19 disease? | \|__\|__\|/ \|__\|__\|__\|/\|__\|__\|__\|__\|  if day or month are not known, write ***99 or 999.*** If year is not known, write ***9999***   Unknown |
| 2.2 | Were you hospitalised? |  Yes   No, skip to Q 2.3 |
| 2.2.1 | If yes, to 2.2, did you receive ventilation or were you admitted to ICU? |  Yes   No   Don’t know |
| 2.3 | Have you experienced/did you experience any of the following symptoms persisting for more than 2 weeks after Covid-19 infection? (*multiple answers possible*) |  Cough, if yes,  productive   Shortness of breath   Breathless under exercise   Chest pain   Fatigue   Numbness in extremities (fingers/feet)   Muscle weakness   Lack of energy   Problems with memory   Poor concentration   Problems with sleep   Anxiety   Loss of taste or smell   None   Other, specify ____________ |
| 2.4 | Do you know what long-Covid or post-Covid-19 condition is? |  Yes, explain _________   No, skip to Q3.1 |
| 2.5 | How did you get information on long-Covid? |  Doctor/healthcare worker told you   Word of mouth from community   Social media   Pamphlets/brochures   Internet   Other, specify ___________ |
| 3.1 | Did you receive any rehabilitation during or after Covid-19? |  Yes   No, skip to Q4.1 |
| 3.2 | If yes, to Q3.1, what rehabilitation in particular did you receive? (*multiple answers possible*) |  Physiotherapy   Occupational therapy   Dietician   Speech therapy and audiology   Psychology   Other, specify ____________ |
| 3.3 | How long after Covid-19 did you start rehabilitation? |  During acute disease   Within 2 weeks   Between 2 to 6 weeks   More than 6 weeks |
| 3.4 | How long was the rehabilitation program? |  Once off   2 weeks to 1 month   1 to 3 months   More than 3 months |
| 3.5 | How frequent did you have visits? |  Weekly or more than once per week   Every two weeks   Once a month   Less frequent than once a month |
| 3.6 | How long did each visit take on average? |  15 minutes or less   15 to 30 minutes   More than 30 minutes |
| 3.7 | How was the rehabilitation carried out? If multiple, select the most used format. |  Face-to-face consultation   Paper-based guidance material or brochure   Phone calls or SMS   Family training   Other, specify _____________ |
| 3.8 | Did you find the rehabilitation helped you? |  Yes, comment how helped ______________________________________   No, comment how did not help ______________________________________ |
| 3.9 | Were you satisfied with the quality of care you received? (*grade on scale 1 to 5*) |  1 – Extremely dissatisfied   2 – Dissatisfied   3 – Neutral   4 – Satisfied   5 – Extremely satisfied |
| 3.10 | What were the biggest challenges you experienced with rehabilitation? (*multiple answers possible*) |  Found the rehabilitation too difficult, specify __   Travel-related costs   Did not have a good relationship with the healthcare worker, or healthcare worker did not understand your needs   Navigating the rehabilitation department and services   Time off work   Unable to practice at home   Did not understand the purpose of the rehabilitation   Did not feel like you were improving   Did not have the resources to conduct rehabilitation (e.g. walking aid)   Lack of family support   Other, specify __________ |
| 3.11 | How could the quality of your rehabilitation be improved? (*multiple answers possible*) |  Longer visit   Shorter waiting times   More frequent visits   Involvement of a family member   Start sessions sooner after infection   More support to raise opinions or goals of rehabilitation   Better educational training on importance or benefit of rehabilitation   Other, specify ___________ |
| 4.1 | If no to Q3.1, would you have liked to receive rehabilitation?  If yes to Q3.1, skip to Q5 |  Yes, why, and what in particular ________   No, why ­­­__________ |
| 4.2 | Do you think rehabilitation would have helped, or would still help, your recovery after Covid-19? |  Yes, why __________   No, why __________ |
| 4.3 | What would be your biggest limitations or challenges when committing to a rehabilitation program? (*multiple answers possible*) |  Transport   Do not understand importance   Scared if something bad happens e.g. strain a muscle   Unable to practice at home   Cannot get time off work   Embarrassed if family or others find out you require assistance   You don’t need it, you are fine   Other, specify ___________ |
| 5. | What are your main goals for recovery  post-Covid-19? (*multiple answers possible*) |  Carry out activities of daily living   Perform exercise   Walk upstairs without resting   Return to work   Be able to care for family   Coping skills   Less social anxiety   Other, specify ___________ |
| 6.1 | What do you think are the benefits of rehabilitation post-Covid-19? |  |
| 6.2 | What do you think the risks are of rehabilitation post-Covid-19? |  |
| 7. | Do you think rehabilitation is an important adjunct for recovery post-Covid-19? |  Yes, explain _________   No, explain _________ |
| 8.1 | Would you find a support group helpful? |  Yes   No |
| 8.2 | If yes to Q8.1, what format would you find the support group helpful? (*multiple answers possible*) |  Facility-based   Social media   Phone calls   Home-based   Other, specify __________ |
| 8.3 | Did you receive any social service support (e.g. grant, food voucher)? |  Yes   No |
| 8.4 | If yes, to Q8.3, which of the following did you receive?  If no, to Q8.3, which of the following do you think would have assisted you? |  Covid-19 grant   Disability grant   Food vouchers   Food parcels   Other, specify ___________ |
| 9. | Any comments |  |

Date completed:

Researcher initials:
