# Supplementary material for: Improving Health and Well-Being of People With Post–COVID-19 Consequences in South Africa: Situation Analysis and Pilot Intervention Design
Source: JMIR Form Res. 2025 Apr 10;9:e58436. doi: 10.2196/58436 (PMC12005461; doi:10.2196/58436)
Supplement: Multimedia Appendix 2 [file formative-v9-e58436-s002.docx]

**Focus Group Discussions Guide**

To get group demographics (name, age, PID, facility) on attendance register.

Welcoming and introductions.

Points to highlight before beginning the discussions:

- Discussing on topics around post-Covid-19 rehabilitation and recovery
- The idea is not to compare your experiences, but to gain insight into the experiences everyone has had, and to appreciate receiving all the information
- Every person is important and please respect each other and what they have to say
- Please raise your hand before speaking, do not interrupt when someone is speaking
- You are welcome to answer in any language you feel comfortable
- The time will be 30-60 minutes, but may take longer depending on how the discussions go
- If we feel you are going off topic, we will ask for you to keep the discussion relevant to this post-Covid topic
- You will not be forced to speak, but we would like for you to interact as much as you feel comfortable
- Should you feel uncomfortable at any point, you can step away from the group or ask to no longer participate
- We do have a counsellor available should you feel the need to speak to a professional
- We will be recording this discussion with a voice recorder and taking notes

Questions:

**Covid-19 experience:**

Can you start by telling us about your Covid-19 experience? What happened and how have you been recovering since? (Probes: long-Covid symptoms, how long ago, severity)

**Covid-19 recovery:**

What support did you have during your recovery from Covid-19? (Probes: specific healthcare provision, any rehabilitation, friend/family support)

What are/were your biggest challenges/concerns? (Probes: transport, lack of motivation, uncertainty, minimal support, poor relationships with healthcare workers)

What were the positives from your recovery experience? (Probes: community support, healthcare worker support, government assistance, any medications, or specific treatments)

What support do you think would have assisted/would assist your recovery more? (Probes: rehab team guidance, family/employer support, physical vs psychological support)

**Future health:**

What are your priorities (and/or concerns) for your health and wellbeing going forward?

**Role of rehabilitation:**

What do you think is the role of rehabilitation in assisting patients’ recovery post-Covid? (Probes: importance, benefits, risks/challenges, where to place amongst other healthcare provision)

What would you value for a potential new intervention for post-Covid-19 rehabilitation? (Probes: economic support, physical exercise training, psychological support)

**Comments:**

Is there anything that we haven’t already covered, that you would like to add or discuss?

Thank everyone for their time.
