# Supplementary material for: Improving Health and Well-Being of People With Post–COVID-19 Consequences in South Africa: Situation Analysis and Pilot Intervention Design
Source: JMIR Form Res. 2025 Apr 10;9:e58436. doi: 10.2196/58436 (PMC12005461; doi:10.2196/58436)
Supplement: Multimedia Appendix 3 [file formative-v9-e58436-s003.docx]

**Clinical Staff Interview Guide**

Points to highlight before beginning interview:

- We will be recording this interview with a voice recorder and taking notes
- Should you feel uncomfortable at any point please let us know and we can stop
- You are free to withdraw at any time
- This interview will take around 30 min but may take longer depending on how the discussion flows
- Please give as much information as you can when answering the questions, allow your thoughts to flow, if we feel you are moving off topic, we will bring you back to base

**Demographics:**

1. Age
2. Gender
3. Years of experience in field
4. Current occupation/title (include department e.g. pulmonologist, physio, OT, psychologist, speech therapist, dietician)
5. Healthcare facility

Questions:

**Covid-19 experience:**

Tell me about your experience working with patients with Covid-19. (Prompts: how did you come to working with Covid patients, what symptomatology/main complaints from patients, number of patients responsible for per day, your role in care)

**Current Covid-19 rehabilitation:**

What rehabilitation programmes are offered for patients with- or post-Covid-19, and how do you come about generating those programmes? (Prompts: methods of assessment and interventions, where do you get guidelines, scheduling visits, multidisciplinary involvement, telemedicine)

What are the difficulties you have experienced managing patients who have had Covid-19? (Prompts: difficulties at facility, difficulties with patients, personal difficulties, lack of support, uncertainty)

What are the benefits or positives you have experienced managing patients who have had Covid-19? (Prompts: patient response, research growth, new interventions, better resource allocation)

**Future Covid-19 rehabilitation:**

What do you think can be done to improve the support for patients who have had Covid? (Prompts: role of rehabilitation, ideas for intervention research)

What would you value for a potential new intervention for post-Covid-19 rehabilitation? (Prompt: economic support, educational training - specify, psychological support)

**Comments:**

Is there anything that we haven’t already covered, that you would like to add or discuss?

Thank you for your time.
